# Coupling Plant Polyphenol Coordination Assembly with Co(OH)_2_ to Enhance Electrocatalytic Performance towards Oxygen Evolution Reaction

**DOI:** 10.3390/nano12223972

**Published:** 2022-11-11

**Authors:** Xue-Zhi Song, Yu-Hang Zhao, Fan Zhang, Jing-Chang Ni, Zhou Zhang, Zhenquan Tan, Xiao-Feng Wang, Yanqiang Li

**Affiliations:** 1 State Key Laboratory of Fine Chemicals, School of Chemical Engineering, Dalian University of Technology, Dalian 116024, China; 2 Key Laboratory of Materials Modification by Laser Ion and Electron Beams, Ministry of Education, School of Physics, Dalian University of Technology, Dalian 116024, China; 3 School of Materials Science and Engineering, North China University of Water Resources and Electric Power, Zhengzhou 450045, China

**Keywords:** Co(OH)_2_, tannic acid, electrocatalyst, OER, coordination assembly

## Abstract

The oxygen evolution reaction (OER) is kinetically sluggish due to the limitation of the four-electron transfer pathway, so it is imperative to explore advanced catalysts with a superior structure and catalytic output under facile synthetic conditions. In the present work, an easily accessible strategy was proposed to implement the plant-polyphenol-involved coordination assembly on Co(OH)_2_ nanosheets. A TA-Fe (TA = tannic acid) coordination assembly growing on Co(OH)_2_ resulted in the heterostructure of Co(OH)_2_@TA-Fe as an electrocatalyst for OER. It could significantly decrease the overpotential to 297 mV at a current density of 10 mA cm^−2^. The heterostructure Co(OH)_2_@TA-Fe also possessed favorable reaction kinetics with a low Tafel slope of 64.8 mV dec^−1^ and facilitated a charge-transfer ability. The enhanced electrocatalytic performance was further unraveled to be related to the confined growth of the coordination assembly on Co(OH)_2_ to expose more active sites, the modulated surface properties and their synergistic effect. This study demonstrated a simple and feasible strategy to utilize inexpensive biomass-derived substances as novel modifiers to enhance the performance of energy-conversion electrocatalysis.

## 1. Introduction

The growing energy and environmental problems caused by the depletion of fossil fuels have led to a desire for clean and sustainable energy [1,2]. The oxygen evolution reaction, as one key reaction in the conversion of electrolytic water to hydrogen [3], nitrogen reduction [4] and metal–air batteries [5], is still limited by the slow four-electron process experienced at the anode [6,7]. In the search for suitable electrocatalysts to reduce the overpotential and speed up the reaction rate, Ru and Ir among the noble metals are preferred, but their high cost and low reserves have prevented us from using them on a large scale [8,9]. Therefore, researchers have turned their attention to transition-metal-based catalysts to find cheaper, better performing alternatives.

Current transition metal-based catalysts include oxides, phosphides, hydroxides, sulfides, high entropy alloys and so on [10,11,12,13,14,15,16]. Hydroxides have a unique structure and redox properties that provide more active sites in the OER process and alter the adsorption energy of the intermediate, thus effectively facilitating electron transport [17]. As a typical transition metal hydroxide, Co(OH)_2_ has both α and β crystalline forms, but its insufficient surface area in and low inherent catalytic ability in each site limits its electrochemical performance [18]. Therefore, the design of catalysts usually starts with the preparation of nanostructures, such as nanosheets or nanoassemblies, to increase the number of electrochemically active sites. The thickness of Co-based lamellar hydroxides has a key influence on its properties [19,20,21]. For example, Duraivel et al. grew layered 3D flower-like Co(OH)_2_ on nickel foam, which greatly enhanced the ion transport rate and enhanced the catalytic efficiency of electrolytic water [22]. In addition, many other approaches have been demonstrated to explore advanced hydroxide-based materials for highly efficient electrocatalysis, such as heteroatom doping and vacancy engineering [23,24]. The creation of heterointerfaces between the hydroxide substrate and functional entity is one vital blueprint to further trigger electrochemical reactions. The heterogeneous interface can synergistically expose more active sites, regulate the electronic structure to promote the intrinsic activity and even enhance the stability, comprehensively optimizing the electrocatalytic OER performance. For instance, noble metal blocks loaded on hydroxides can modulate the oxidation states of a transition metal center, tune the adsorption ability of intermedia to strengthen per-site electrocatalytic activity and even change the catalytic mechanism to interfacial direct O-O coupling, as demonstrated by Yamauchi and our group [25,26,27]. Moreover, metal–organic coordination units, usually researched as MOFs, have been integrated with hydroxide to heterostructures for efficient electrocatalysis due to their adjustable composition and interfacial interaction [28,29,30].

As one important coordination member, metal–polyphenol coordination networks have recently attracted much attention in electrocatalysis. Tannic acid, a natural polyphenol derived from plants, is inexpensive and environmentally friendly, and its numerous hydroxyl groups allow it to be assembled with a variety of metal ions [31]. Most of the substances formed by tannic acid with metal ions are amorphous and can expose more active sites, thus enhancing electrocatalytic activity [32,33]. A number of assemblies of tannic acid with metal ions have been reported, using surface engineering strategies to modify the interfacial and surface properties of non-homogeneous catalysts to significantly improve the catalytic activity of water electrolysis [34,35]. At the same time, the high affinity of tannic acid for the substrate enables the assembly of small and dispersed nanoalloy particles through the abundance of functional groups [36,37]. Therefore, it can be deemed that the assembly of one metal–polyphenol coordination unit on another hydroxide material will lead to superior electrocatalytic performance for the OER.

In this work, we developed a simple and rapid synthetic method to prepare TA-Fe complexes engineered on Co(OH)_2_, significantly decreasing the overpotential to 297 mV to achieve a current density of 10 mA cm^−2^ and possessing a small Tafel slope of 64.8 mV dec^−1^. This easily synthesized route in an aqueous solution under an ambient environment could provide significant prospective to develop coordination-assembly-involved heterostructures for efficient OER electrocatalysis.

## 2. Materials and Methods

### 2.1. Materials

Cobalt nitrate hexahydrate (Co(NO_3_)_2_·6H_2_O), cetyltrimethylammonium bromide (CTAB), potassium hydroxide (KOH) and ferric chloride hexahydrate (FeCl_3_·6H_2_O) were provided by Tianjin Damao Chemical Reagent corporation. Tannic acid (TA) and sodium borohydride (NaBH_4_) were purchased from Aladdin. Nafion solution (5 wt%) was purchased from the Aldrich corporation. All chemicals were used directly without further purification. In addition to the above, carbon paper was purchased from the Jinglong Special Carbon Company, Beijing, China.

### 2.2. Synthesis of Co(OH)_2_

Co(NO_3_)_2_·6H_2_O (2 mmol, 0.582 g) and 0.5 g of CTAB were dissolved in deionized water (25 mL) and stirred for 30 min to obtain homogeneous solution A. Then, NaBH_4_ (0.1 g) was dissolved in 10 mL of deionized water to obtain solution B. The as-prepared solution B was added to solution A drop by drop. When the color of the reaction mixture had completely changed from black to green, the product of Co(OH)_2_ was collected by centrifugation and washed three times with deionized water and ethanol. Finally, the products were placed in an oven at 60 °C for drying.

### 2.3. Synthesis of Co(OH)_2_@TA-Fe

The as-prepared Co(OH)_2_ (20 mg) sample was ultrasonically dispersed in 5 mL of deionized water. Tannic acid (81.6 mg) was dissolved in deionized water (2 mL) and 1 M KOH (160 μL) was added dropwise. A total of 200 μL of the configured tannic acid (TA) solution was added to the Co(OH)_2_ suspension and stirred for 10 min, and then 7.47 mg of FeCl_3_·6H_2_O was added and continuously stirred for two hours. In addition, the product was finally collected by centrifugation, washed three times with deionized water and ethanol and then placed in an oven to dry. The product was denoted as Co(OH)_2_@TA-Fe.

### 2.4. Synthesis of Co(OH)_2_@TA, Co(OH)_2_@Fe and TA-Fe

The preparation process of Co(OH)_2_@TA-Fe was followed to prepare Co(OH)_2_@TA and TA-Fe, except that FeCl_3_·6H_2_O or Co(OH)_2_ was not present. The sample obtained under the addition of 10 μL of 1 M KOH without the addition of tannic acid was named as Co(OH)_2_@Fe.

### 2.5. Materials Characterization

The morphological characterization was performed by field emission scanning electron microscopy (FESEM, NovaSEM 450) with an accelerating voltage of 18 kV and transmission electron microscopy (TEM, FEI Tecnai G2 F30). The crystal structure was characterized by X-ray powder diffraction (XRD, XRD-7000S), the tests were performed under Cu-Kα (λ = 1.5406 Å) radiation and the scans were performed at 5° min^−1^ in the range of 5–80°. In addition, the surface analysis of the samples was performed by Fourier transform infrared spectroscopy (FT-IR, Nicolet iN10) and X-ray photoelectron spectroscopy (XPS, ESCALABTM 250Xi) manufactured by ThermoFisher, Waltham, MA, USA. ICP testing was conducted on a 7900X model from Agilent.

### 2.6. Electrochemical Measurements

All electrochemical measurements were performed at room temperature using a CHI660E workstation with a three-electrode system from C&H Instruments, Shanghai. The electrolysis cell made of quartz had a capacity of 300 mL and was equipped with a Teflon cover (Tianjin Aida Hengsheng Technology Development Co., Ltd., Tianjin, China). A glassy carbon electrode loaded with catalyst was used as the working electrode, a carbon rod as the counter electrode and Hg/HgO as the reference electrode to form the three-electrode system. A total of 1 M KOH was used as the electrolyte. Catalyst ink was obtained by ultrasonically dispersing 4 mg of catalyst in 480 µL of deionized water, 480 µL of anhydrous ethanol and 40 µL of Nafion solution. A total of 16 μL of catalyst ink was added to the glassy carbon electrode in two drops. The loading of catalyst on the glassy carbon electrode was 0.326 mg cm^−2^. The data were transformed relative to a reversible hydrogen electrode (RHE) using the following Equation:

*E*_RHE_ = *E*_Hg/HgO_ + 0.0591 ∗ pH + 0.098 V (pH = 13.98) (1)

Linear sweep voltammetry (LSV) curves were obtained at a scan rate of 5 mV s^−1^. Cyclic voltammetry (CV) curves were obtained using different scan rates of 20–100 mV s^−1^ at a voltage range of 0.05 to 0.15 V (vs. *E*_Hg/HgO_) and the double-layer capacitance (*C*_dl_) calculation was conducted. Electrochemical impedance spectroscopy (EIS) measurements were taken over a frequency range of 0.01–10^5^ Hz (AC amplitude: 5 mV) from an overpotential of 297 mV. Stability tests were performed with the catalyst (1 mg cm^−2^) loaded on carbon paper.

## 3. Results and Discussion

**Figure 1:**
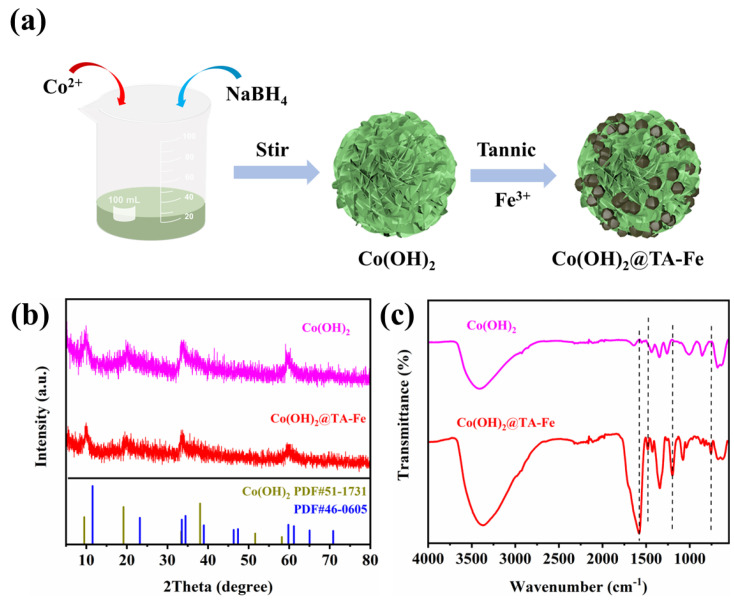
(**a**) Schematic image of the preparation route of Co(OH)_2_@TA−Fe. (**b**) XRD patterns and (**c**) FT−IR spectra of Co(OH)_2_ and Co(OH)_2_@TA−Fe.

The route used to fabricate Co(OH)_2_@TA-Fe is shown in Figure 1a. Firstly, a dense nanosheet network structure of Co(OH)_2_ was prepared by the surfactant-assisted simultaneous reduction−hydrolysis method [38]. The Co(OH)_2_ material was utilized as a template to grow TA-Fe complexes on the surface, leading to the formation of Co(OH)_2_@TA-Fe heterostructures.

The crystallinity of the as-synthesized materials was confirmed by powder XRD, as shown in Figure 1b. In the PXRD pattern of pristine Co(OH)_2_, distinct diffraction peaks at two-theta values of about 9.5 and 19.1° could be indexed to the (001) and (002) crystal planes of Co(OH)_2_ (JCPDS No. 51-1731), and extra diffraction peaks at 33.5, 34.5, 59.8 and 70.8° corresponded to the (100), (102), (110) and (202) crystalline planes, respectively (JCPDS No. 46-0605). When tannic acid was chelated with Fe^3+^ ions on the surface, the XRD pattern showed no change. This revealed that the coordination assembly did not change the basic structure of the original Co(OH)_2_ and the metal–phenol coordination compound was amorphous. [39,40]. In the FT-IR spectrum of Co(OH)_2_@TA-Fe, the absorption peaks at 3415, 2922 cm^−1^ were attributed to aromatic O-H stretching vibration and C-H stretching, respectively. The peaks at 1575, 1484 and 1204 cm^−1^ belonged to aromatic C=C and phenolic C-O stretching in TA anions compared to the pure Co(OH)_2_. The successful coordinative assembling of the complex between the metallic Fe^3+^ ions and tannic acid on Co(OH)_2_ could be demonstrated by the above. For comparison, samples were made without the addition of tannic acid or FeCl_3_·6H_2_O, or without the addition of Co(OH)_2_ precursors, and the effect of tannic acid addition on the catalyst was also compared. After the ICP test, it was found that the Co and Fe content of the Co(OH)_2_@TA-Fe samples were 30.41 and 4.01% (wt%), respectively (Table S1).

**Figure 2:**
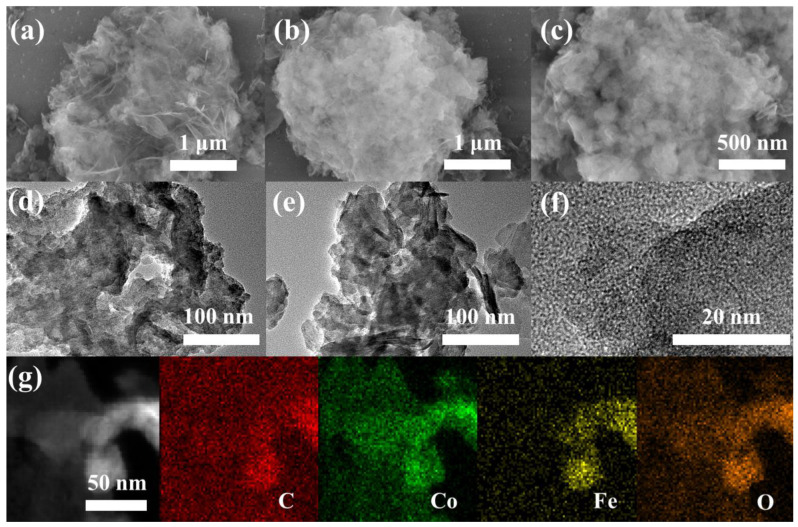
SEM images (**a**–**c**), TEM images (**d**,**e**), HRTEM images (**f**) and elemental mapping (**g**) of Co(OH)_2_@TA-Fe.

The morphologies were characterized by scanning electron microscopy. As shown in Figure S1, the Co(OH)_2_ was formed by a large number of cross-linking nanosheets, which could provide sufficient regions to attract exogenous components for assembly to form heterostructures. The co-addition of tannic acid and FeCl_3_·6H_2_O led to the formation of a large number of metal-TA particles on the surface (Figure 2a–c). In addition, comparing Co(OH)_2_@TA with Co(OH)_2_@Fe together, the combination of individual tannic acid or FeCl_3_ with Co(OH)_2_ did not destroy the overall nano-flake morphology of the Co(OH)_2_ (Figures S2 and S3). If the tannic acid was coordinated with FeCl_3_·6H_2_O directly in the absence of a Co(OH)_2_ substrate, irregular large particles were produced (Figure S4). This striking morphological difference suggested the confined growth of the coordination assembly on Co(OH)_2_. Further observation of the detailed structure of the Co(OH)_2_@TA-Fe using transmission electron microscopy (TEM) revealed that the Co(OH)_2_ nanosheets were wrapped with many TA-Fe nanoparticles (Figure 2d,e), which was consistent with the SEM observations. There were no obvious lattice stripes on the high-resolution TEM images, demonstrating the formation of an amorphous phase of TA-Fe particles (Figure 2f). In the elemental mapping images, a uniform distribution of C, Co, Fe and O elements was observed (Figure 2g). According to the EDS elemental analysis, the weight ratios of Fe and Co were 4.1% and 24.19% (wt%), respectively, which were close to the results obtained from the ICP testing (Figure S5 and Table S2).

**Figure 3:**
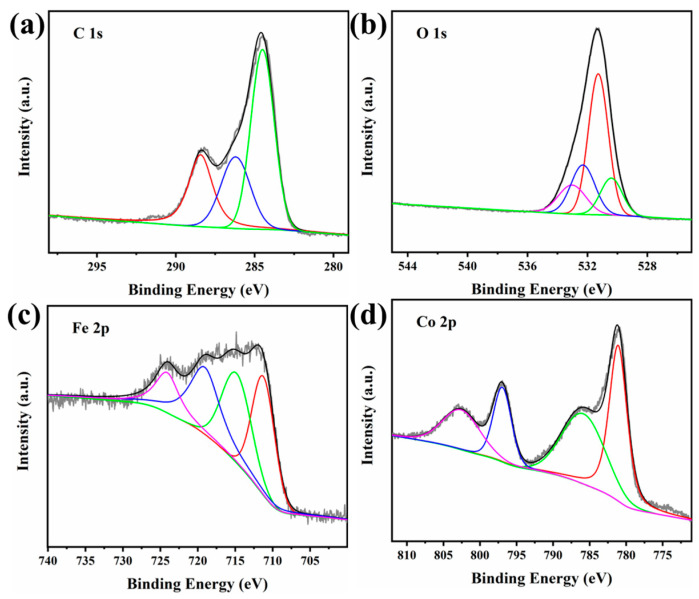
XPS spectra of Co(OH)_2_@TA-Fe: C 1s (**a**), O 1s (**b**), Fe 2p (**c**) and Co 2p (**d**).

The elemental chemical bonding states of the Co(OH)_2_@TA-Fe were investigated by X-ray photoelectron spectroscopy (Figure S6). Figure 3a shows the XPS spectra of element C. The binding energy peaks at 284.5, 286.2 and 288.4 eV belonged to C-C, C-O and C=O [41], respectively, while in the XPS spectrum of O, the binding energy located at 530.4 eV was attributed to metal-O bonding, and 531.3, 532.3 and 533.2 eV were attributed to C=O, O-H and C-O, respectively [42,43]. The XPS spectra of Fe showed that 711.2, 714.8 and 724.1 eV belonged to Fe^3+^ and 719.1 eV was the satellite peak of Fe^3+^ (Figure 3c) [44,45]. Finally, the XPS of Co was split into 781.1 and 796.9 eV attributed to Co^2+^ and 785.8 and 802.6 eV attributed to their satellite peaks, which were not quite different from the XPS of the Co(OH)_2_ (Figure S7) [46]. The above analyses further proved the formation of the TA-Fe complex and its successful attachment on Co(OH)_2_. The weight of Fe and Co for the Co(OH)_2_@TA-Fe obtained by XPS calculations were 13.9% and 26.36% (wt%) (Table S3). The large difference in the contents determined by XPS and other methods may be due to the structure that the TA-Fe loaded on the Co(OH)_2_ surface. Therefore, the amount of iron determined on the surface was larger than that determined by other methods, since XPS is a surface-sensitive analytical method.

**Figure 4:**
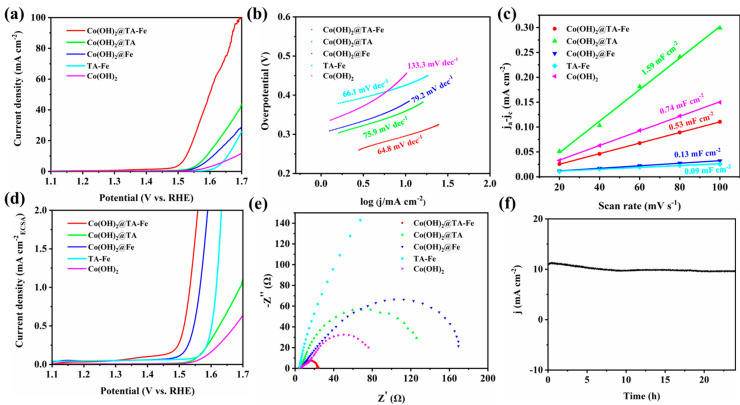
(**a**) LSV curves, (**b**) Tafel slopes, (**c**) *C*_dl_ calculations, (**d**) LSV curve normalized by ECSA and (**e**) Nyquist plots for Co(OH)_2_@TA−Fe, Co(OH)_2_@Fe, TA−Fe, Co(OH)_2_@TA and Co(OH)_2_. (**f**) Long−term stability of Co(OH)_2_@TA−Fe.

The catalysts were tested for their OER electrocatalytic performance using the assembled three-electrode system in 1 M KOH solution. As shown in Figure 4a, the linear sweep voltammetry curves of all samples were not iR compensated. The overpotential of the Co(OH)_2_@TA-Fe sample was 297 mV with respect to the reference current density of 10 mA cm^−2^. This overpotential of 297 mV was much lower than that of the Co(OH)_2_@TA (361 mV), Co(OH)_2_@Fe (381 mV), TA-Fe (427 mV) and Co(OH)_2_ (455 mV). This demonstrated that compounding TA-Fe particles with Co(OH)_2_ substantially improved the electrocatalytic activity of the catalysts. The overpotential of the Co(OH)_2_@TA-Fe increased when the amount of feeding tannic acid increased (Figure S8), proving that the formation of more TA-Fe complexes did not lead to further performance enhancement of the samples. Samples with different feeding Fe amounts were also prepared, and it was again proved that Co(OH)_2_@TA-Fe with a proper amount of source Fe possessed excellent electrocatalytic properties (Figure S9). Moreover, the Tafel slope of the Co(OH)_2_@TA-Fe (64.8 mV dec^−1^) was lower than that of the Co(OH)_2_@TA (75.9 mV dec^−1^), Co(OH)_2_@Fe (79.2 mV dec^−1^), TA-Fe (66.1 mV dec^−1^) and Co(OH)_2_ (133.3 mV dec^−1^), suggesting its faster intrinsic reaction kinetics enabled by the Co(OH)_2_@TA-Fe heterostructure. Compared with many recently reported catalysts (Table S4), such as Co_3−x_Fe_x_O_4_ (294 mV, 47.3 mV dec^−1^) [47], MPN@Fe_3_O_4_ (260 mV, 33.6 mV dec^−1^) [48], CoFe(OH)_x_/GO (294 mV, 63.1 mV dec^−1^) [49], TF@Co(OH)_2_-500 (317 mV, 47 mV dec^−1^) [50] and CeO_2_@Co(OH)_2_ (310 mV, 66 mV dec^−1^) [51], the Co(OH)_2_@TA-Fe had a comparable or even better catalytic performance.

To further disclose the inherent reason for the impressive electrocatalytic activity, the electrochemical surface area (ECSA) of the electrocatalyst was estimated by electrochemical double-layer capacitance (*C*_dl_) using cyclic voltammetry (CV) in the non-Faraday current region at different scan rates (Figure S10). As shown in Figure 4c, the *C*_dl_ values for the Co(OH)_2_@TA-Fe (0.53 mF cm^−2^) were larger than those for the Co(OH)_2_@Fe (0.13 mF cm^−2^) and TA-Fe (0.09 mF cm^−2^), but smaller than those for the Co(OH)_2_@TA (1.59 mF cm^−2^) and Co(OH)_2_ (0.74 mF cm^−2^). ECSA values can be obtained from *C*_dl_, which can help to understand the effect between surface roughness in terms of morphology and improvement in terms of catalytic performance. At reaching an ECSA-normalized current density of 1 mA cm^−2^ (Figure 4d), the Co(OH)_2_@TA-Fe only required 1.534 V compared to the Co(OH)_2_@TA (1.692 V), Co(OH)_2_@Fe (1.565 V) and TA-Fe (1.617 V). These compared results implied that the heterostructure with a coordination assembly on the Co(OH)_2_ could enhance the intrinsic per-site activity towards the OER. In addition, the EIS values of the different samples were measured to further characterize the charge-transfer ability at the electrode/electrolyte interface during OER catalysis. As shown in the Nyquist plot (Figure 4e), the Co(OH)_2_@TA-Fe catalyst had the smallest radius among these five electrocatalysts and exhibited the smallest charge-transfer resistance, demonstrating that combining TA-Fe with Co(OH)_2_ could effectively reduce the charge-transfer impedance and enhance the OER activity by increasing the charge-transfer rate. Long-term stability is another important factor in judging the performance of a catalyst. As shown in Figure 4f, after continuously applying a bias for 24 h, the current density of the Co(OH)_2_@TA-Fe catalyst showed almost a straight line with negligible attenuation, further demonstrating its excellent electrochemical stability.

**Figure 5:**
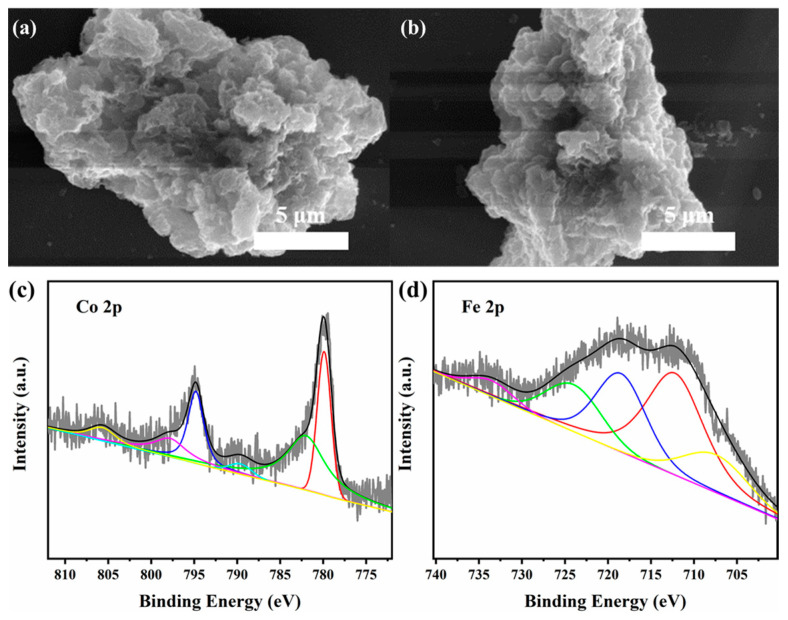
(**a**,**b**) SEM images of Co(OH)_2_@TA-Fe that had undergone stability test and XPS spectra of Co(OH)_2_@TA-Fe after stability test: (**c**) Co 2p region, (**d**) Fe 2p region.

In the SEM image of the Co(OH)_2_@TA-Fe sample after the stability test (Figure 5a,b), no obvious nanosheet structure could be observed. In contrast, there were agglomerated bulk particles. The corresponding XPS analysis was also performed. The Co 2p 3/2 peak showed a negative shift from 781.1 to 779.9 eV, indicating a conversion from Co^2+^ to Co^3+^ after OER catalysis (Figure 5c) [52]. Satellite peaks at 707.2 eV attributed to Fe^0^ and 733.5 eV attributed to Fe^2+^ were also observed (Figure 5d) [53,54]. The presence of Fe^0^ could increase the conductivity and promote the appearance of CoOOH material that is favorable to OER [55]. In addition, the metal phenolic network formed by the tannic acid and Fe could prevent the loss of Fe and further enhance the stability of the catalyst. Therefore, its excellent OER performance allows Co(OH)_2_@TA-Fe to be a promising anodic candidate for water electrolysis.

In combination with the above experimental analysis, the enhanced electrocatalytic performance of the Co(OH)_2_ modified by the complexation of tannic acid with iron could be attributed to the following points: (i) Co(OH)_2_ nanosheets could be used as a substrate with a large specific surface area to shorten the ion and electron diffusion pathways; (ii) using Co(OH)_2_ as a substrate could prevent the tannic acid from complexing with metal ions in large quantities to form bulk particles, which could expose more active sites; (iii) water molecules are more favorable to adsorb on Fe^3+^ than Co^2+^ [56], and the TA-Fe could change the surface properties of the catalyst; and v) this synergistic effect of heterogeneous structure.

## 4. Conclusions

In summary, Co(OH)_2_@TA-Fe catalysts were successfully demonstrated by rational surface engineering with TA-Fe coordination units on the surface of Co(OH)_2_ nanosheets. The confined growth of exogenous assemblies with amorphous features provided more active sites and simultaneously enriched the interfaces. The heterostructured coordination assembly could significantly enhance the electrocatalytic activity with only 297 mV of Co(OH)_2_@TA-Fe required to reach 10 mA cm^−2^. Moreover, it also induced more favorable reaction kinetics and improved the charge-transfer ability and intrinsic activity of the catalytic site. This work can open the way for fabricating multi-phase environmentally friendly and cheap catalysts towards energy conversion, in which the size, crystallinity and metal type of the metal–polyphenol entity may be designed to enhance the performance.

## Data Availability

Not applicable.

## Supplementary Materials

The following are available online at https://www.mdpi.com/article/10.3390/nano12223972/s1, Figure S1: SEM images of Co(OH)_2_; Figure S2: SEM images of Co(OH)_2_@TA; Figure S3: SEM images of Co(OH)_2_@Fe; Figure S4: SEM images of TA-Fe; Figure S5. EDS analysis graph of Co(OH_)2_@TA-Fe. Figure S6: Full XPS spectrum of Co(OH)_2_@TA-Fe; Figure S7: XPS spectra of Co(OH)_2_; Figure S8: LSV curves for Co(OH)_2_@TA-Fe with different amounts of tannic acid added; Figure S9. Comparison of LSV performance tests with different Fe additions. Figure S10: CV curves of different samples; Table S1. ICP test results of Co(OH)_2_@TA-Fe. Table S2. Results of EDS elemental analysis of Co(OH)_2_@TA-Fe. Table S3. Surface composition of the Co(OH)_2_@TA-Fe obtained by XPS measurements. Table S4: Comparison of electrocatalytic performance with other catalysts. References [29,48,49,50,51,57,58,59,60,61,62,63] are cited in the Supplement Material.
