# Supplementary material for: Coupling Plant Polyphenol Coordination Assembly with Co(OH)2 to Enhance Electrocatalytic Performance towards Oxygen Evolution Reaction
Source: Nanomaterials (Basel). 2022 Nov 11;12(22):3972. doi: 10.3390/nano12223972 (PMC9699349; doi:10.3390/nano12223972)
Supplement: Supplementary file 1 [file nanomaterials-12-03972-s001.zip › nanomaterials-2010070-supplementary.pdf]

Supporting Information

Coupling Plant Polyphenol Coordination Assembly with $Co(OH)_2$ to Enhance Electrocatalytic Performance towards Oxygen Evolution Reaction

Xue-Zhi Song,[a] Yu-Hang Zhao,[a] Fan Zhang,[a] Jing-Chang Ni,[a] Zhou Zhang,[a] Zhenquan Tan,[a] Xiao-Feng Wang[*,b] and Yanqiang Li[*,c]

[a]State Key Laboratory of Fine Chemicals, School of Chemical Engineering, Dalian University of Technology, Dalian 116024, China;

[b]Key Laboratory of Materials Modification by Laser Ion and Electron Beams, Ministry of Education, School of Physics, Dalian University of Technology, Dalian, 116024, China.

[c] School of Materials Science and Engineering, North China University of Water Resources and Electric Power, Zhengzhou, 450045, China E-mail: lyqncwu@126.com

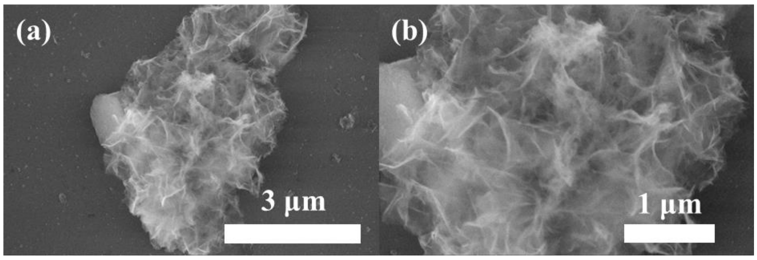

Figure S1. SEM images of $Co(OH)_2$.

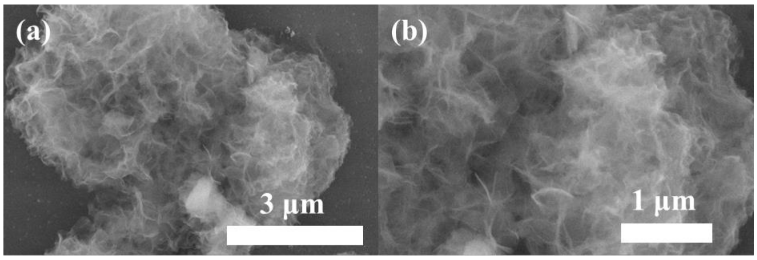

Figure S2. SEM images of $Co(OH)_2$@TA.

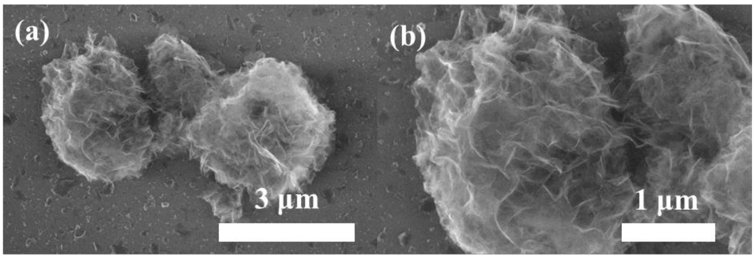

Figure S3. SEM images of $Co(OH)_2$@Fe.

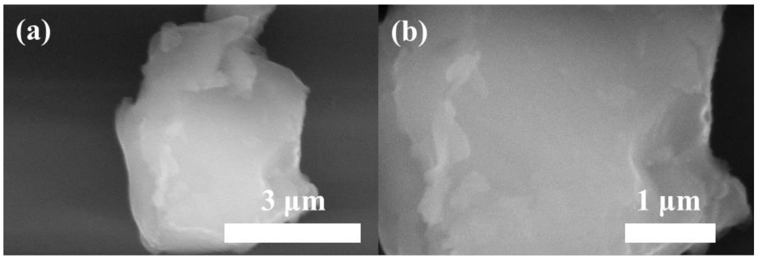

Figure S4. SEM images of TA-Fe.

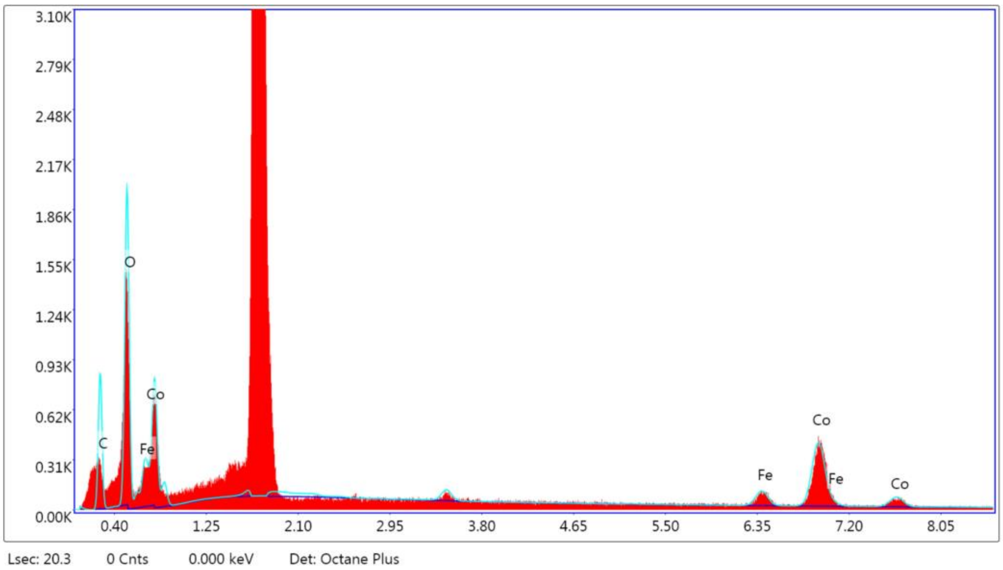

Figure S5. EDS analysis graph of $Co(OH)_2$@TA-Fe.

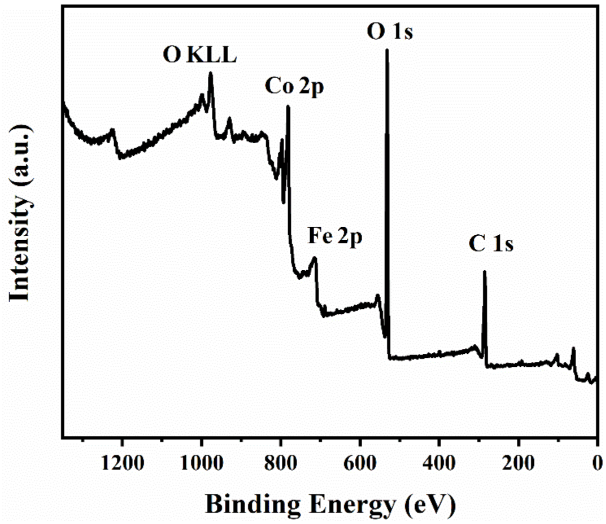

Figure S6. Full XPS spectrum of $Co(OH)_2$@TA-Fe.

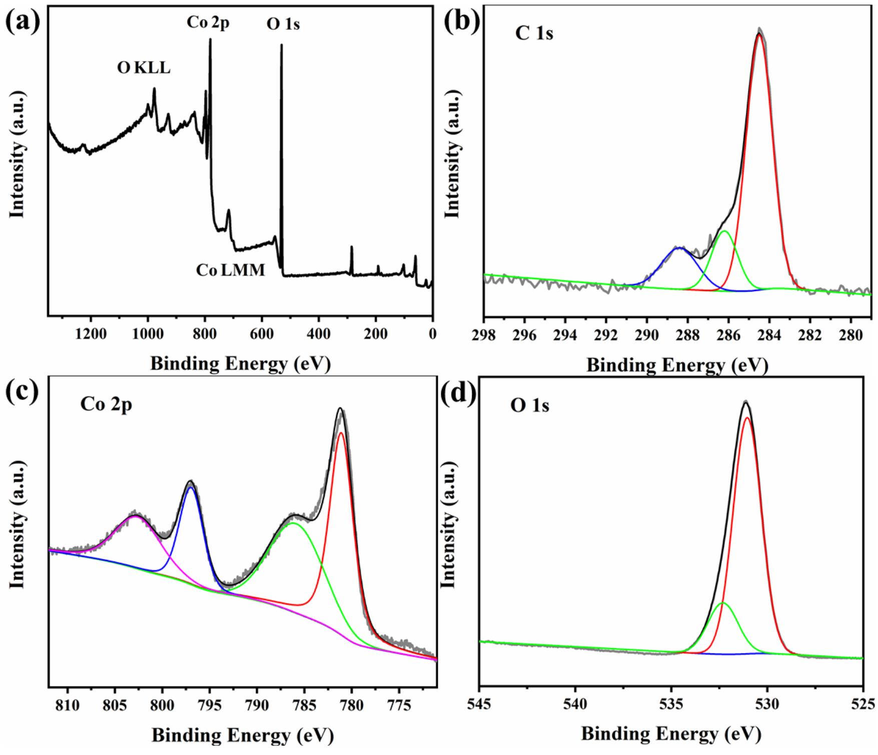

Figure S7. XPS spectra of $Co(OH)_2$.

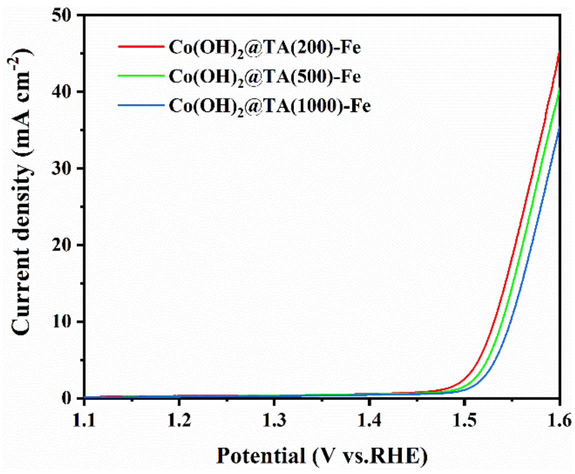

Figure S8. LSV curves for $Co(OH)_2$@TA-Fe with different amount of tannic acid added.

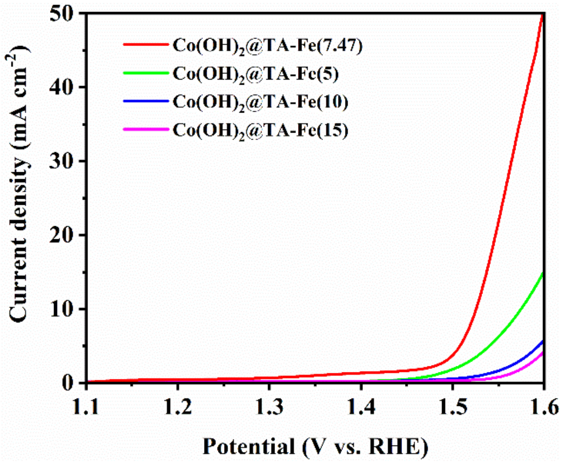

Figure S9. Comparison of LSV performance tests with different Fe additions.

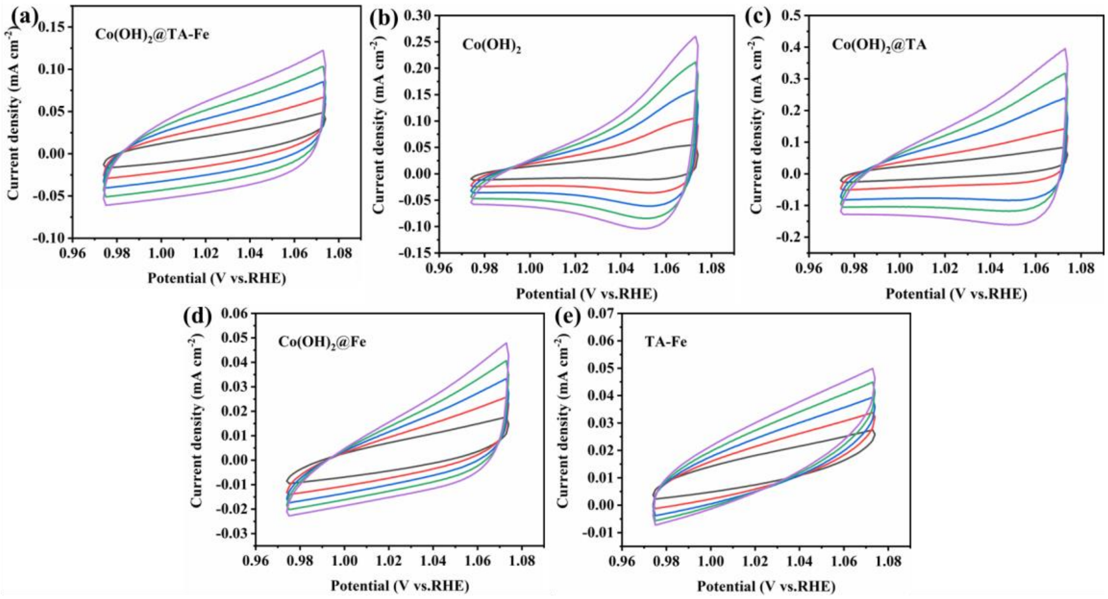

Figure S10. CV curves of different samples.

Table S1. ICP test results of $Co(OH)_2$@TA-Fe

Sample	Co(wt.%)	Fe(wt.%)	Molar ratio of Fe and Co
$Co(OH)_2$@TA-Fe	30.41	4.01	0.125

Table S2. Results of EDS elemental analysis of $Co(OH)_2$@TA-Fe.

Element	Weight (%)	Atomic (%)
C K	28.56	42.77
O K	43.16	48.53
Fe K	4.1	1.32
Co K	24.19	7.38

Table S3. Surface composition of the $Co(OH)_2$@TA-Fe obtained by XPS measurements.

C(wt.%)	O(wt.%)	Co(wt.%)	Fe(wt.%)
25.32	34.39	26.36	13.9

Table S4. Comparison of electrocatalytic performance with other catalysts

Catalyst	Electrolyte	Overpotential ($10\ mV\ cm^{-2}$)	Tafel ($mV\ dec^{-1}$)	Reference
$Co(OH)_2$@TA-Fe	1.0 M KOH	297	64.8	This work
$CoSe/Co(OH)_2$	1.0 M KOH	299	91	[1]
MPN@Fe_3O_4	1.0 M KOH	260	33.6	[2]
$Co(OH)_2$@$Ni(OH)_2$/CC	1.0 M KOH	330	223	[3]
$CoFe(OH)_x$/GO	1.0 M KOH	294	63.4	[4]
TF@$Co(OH)_2$-500	1.0 M KOH	317	47	[5]
Fe_1Co_2-NC	1.0 M KOH	356	86.6	[6]
$CoC_x/(Co_{0.55}Fe_{1.945})_2P$@C	1.0 M KOH	390	65	[7]
CoFeV	1.0 M KOH	376	25.6	[8]
CoFe-NCNFs	1.0 M KOH	323	63.9	[9]
CoFe-Co@PNC	1.0 M KOH	320	81	[10]
$Co(OH)_2$@NC	1.0 M KOH	330	79	[11]
CeO_2@$Co(OH)_2$	1.0 M KOH	310	66	[12]

References

1. Gong, C.; Li, W.; Lei, Y.; He, X.; Chen, H.; Du, X.; Fang, W.; Wang, D.; Zhao, L. Interfacial engineering of ZIF-67 derived CoSe/$Co(OH)_2$ catalysts for efficient overall water splitting. *Compos. Part B Eng.* **2022**, *236*, 109823.
2. Jia, X.; Wu, J.; Lu, K.; Li, Y.; Qiao, X.; Kaelin, J.; Lu, S.; Cheng, Y.; Wu, X.; Qin, W. Organic–inorganic hybrids of Fe–Co polyphenolic network wrapped Fe_3O_4 nanocatalysts for significantly enhanced oxygen evolution. *J. Mater. Chem. A* **2019**, *7*, 14302–14308.
3. Wang, Y.; He, Y.; Zhou, M. Fabrication of hierarchical $Co(OH)_2$@$Ni(OH)_2$ core-shell nanosheets on carbon cloth as an advanced electrocatalyst for oxygen evolution reaction. *Appl. Surf. Sci*. **2019**, *479*, 1270–1276.
4. Cheng, J.; Yue, X.; Chen, C.; Shen, X.; Zeng, S.; Ji, Z.; Yuan, A.; Zhu, G. Template-assisted synthesis of accordion-like CoFe(OH) nanosheet clusters on GO sheets for electrocatalytic water oxidation. *J. Electroanal. Chem*. **2022**, *905*, 115957.
5. Zhou, Y.N.; Fan, R.Y.; Cao, Y.N.; Wang, H.Y.; Dong, B.; Zhao, H.Y.; Wang, F.L.; Yu, J.F.; Chai, Y.M. Oriented and robust anchoring of Fe via anodic interfacial coordination assembly on ultrathin Co hydroxides for efficient water oxidation. *Nanoscale* **2021**, *13*, 13463–13472.
6. Lei, Y.; Huang, R.; Xie, H.; Zhang, D.; Liu, X.; Si, Y.; Li, N. Electronic structure tuning of FeCo nanoparticles embedded in multi-dimensional carbon matrix for enhanced bifunctional oxygen electrocatalysis. *J. Alloys Compd*. **2021**, *853*, 157070.
7. Wu, Y.; Xiao, Z.; Jin, Z.; Li, X.; Chen, Y. The cobalt carbide/bimetallic CoFe phosphide dispersed on carbon nanospheres as advanced bifunctional electrocatalysts for the ORR, OER, and rechargeable Zn-air batteries. *J. Colloid Interface Sci*. **2021**, *590*, 321–329.
8. Adamson, W.; Jia, C.; Li, Y.; Zhao, C. Vanadium-induced fragmentation of crystalline CoFe hydr(oxy)oxide electrocatalysts for enhanced oxygen evolution reaction. *Int. J. Hydrog. Energy* **2021**, *46*, 35230–35238.
9. Lin, S.-Y.; Chen, Y.-P.; Cao, Y.; Zhang, L.; Feng, J.-J.; Wang, A.-J. Aminouracil-assisted synthesis of CoFe decorated bougainvillea-like N-doped carbon nanoflowers for boosting Zn–air battery and water electrolysis. *J. Power Sources* **2022**, *521*, 230926.
10. Lei, Z.; Tan, Y.; Zhang, Z.; Wu, W.; Cheng, N.; Chen, R.; Mu, S.; Sun, X. Defects enriched hollow porous Co-N-doped carbons embedded with ultrafine CoFe/Co nanoparticles as bifunctional oxygen electrocatalyst for rechargeable flexible solid zinc-air batteries. *Nano Res.* **2020**, *14*, 868–878.
11. Li, G.; Liu, C.; Zhang, Z.; Cui, B.; Chen, Y.; Deng, Y.; Hu, W. Nano-manufacturing of $Co(OH)_2$@NC for efficient oxygen evolution/reduction reactions. *J. Mater. Sci. Technol.* **2021**, *81*, 131–138.
12. Sung, M.-C.; Lee, G.-H.; Kim, D.-W. CeO_2/$Co(OH)_2$ hybrid electrocatalysts for efficient hydrogen and oxygen evolution reaction. *J. Alloys Compd*. **2019**, *800*, 450–455.
